# Use of poly ADP-ribose polymerase [PARP] inhibitors in cancer cells bearing DDR defects: the rationale for their inclusion in the clinic

**DOI:** 10.1186/s13046-016-0456-2

**Published:** 2016-11-24

**Authors:** Aniello Cerrato, Francesco Morra, Angela Celetti

**Affiliations:** IEOS, CNR, via S. Pansini 5, 80131 Naples, Italy

**Keywords:** DNA damage response, PARP enzymes, PARP inhibitors, Cancer, BRCA1/2 and BRCAness, Clinical trials, Assays, HR proficiency and PARP activity

## Abstract

**Background:**

DNA damage response (DDR) defects imply genomic instability and favor tumor progression but make the cells vulnerable to the pharmacological inhibition of the DNA repairing enzymes. Targeting cellular proteins like PARPs, which cooperate and complement molecular defects of the DDR process, induces a specific lethality in DDR defective cancer cells and represents an anti-cancer strategy. Normal cells can tolerate the DNA damage generated by PARP inhibition because of an efficient homologous recombination mechanism (HR); in contrast, cancer cells with a deficient HR are unable to manage the DSBs and appear especially sensitive to the PARP inhibitors (PARPi) effects.

**Main body:**

In this review we discuss the proof of concept for the use of PARPi in different cancer types and the success and failure of their inclusion in clinical trials.

The PARP inhibitor Olaparib [AZD2281] has been approved by the FDA for use in pretreated ovarian cancer patients with defective BRCA1/2 genes, and by the EMEA for maintenance therapy in platinum sensitive ovarian cancer patients with defective BRCA1/2 genes. BRCA mutations are now recognised as the molecular targets for PARPi sensitivity in several tumors. However, it is noteworthy that the use of PARPi has shown its efficacy also in non-BRCA related tumors. Several trials are ongoing to test different PARPi in different cancer types. Here we review the concept of BRCAness and the functional loss of proteins involved in DDR/HR mechanisms in cancer, including additional molecules that can influence the cancer cells sensitivity to PARPi. Given the complexity of the existing crosstalk between different DNA repair pathways, it is likely that a single biomarker may not be sufficient to predict the benefit of PARP inhibitors therapies. Novel general assays able to predict the DDR/HR proficiency in cancer cells and the PARPi sensitivity represent a challenge for a personalized therapy.

**Conclusions:**

PARP inhibition is a potentially important strategy for managing a significant subset of tumors. The discovery of both germline and somatic DNA repair deficiencies in different cancer patients, together with the development of new PARP inhibitors that can kill selectively cancer cells is a potent example of targeting therapy to molecularly defined tumor subtypes.

## Methodology: sources and search terms

Literature from a range of sources, including PubMed and MEDLINE, were searched to identify recent reports regarding “DNA damage repair and PARP inhibitors” in addition to other terms relevant to this Review, including “Breast cancer and PARP”, “synthetic lethality”, “cancer and PARP inhibitors”, and “BRCAness”. The reference lists of key articles identified were also searched for additional relevant publications. The ClinicalTrials.gov database was searched using the term “PARP inhibitors” to identify relevant clinical trials.

The key points of this review are:

- # The poly(ADP-ribose) polymerases (PARPs) family.
- # Repair of single-strand and double-strand breaks in DNA damage.
- # Homologous recombination repair (HRR) mechanisms.
- # Defects in DNA Damage Response in cancer.
- # BRCA1 or BRCA2 mutations.
- # Synthetic lethal concept
- # Molecular defects which cause the lack of homologous recombination and produce sensitivity to inhibitors of PARP activity.
- # Chromosomal instability and DNA repair foci
- # in vitro and ex vivo assays to predict the efficacy of PARP inhibitors.
- # Success and failure of PARP inhibitors in Clinical Trials.

## Background

DNA damage response (DDR) is the cellular reaction to exogenous and endogenous genotoxic injuries that may produce DNA single strand breaks (SSBs) and DNA double strand breaks (DSBs). While SSBs are repaired by mechanisms of nucleotide excision repair (NER) or base excision repair (BER), or mismatch repair (MMR), DSBs are repaired either by the mechanism of homologous recombination (HR), which utilizes the sister chromatid as a template for a correct replacement of the DNA sequence, or by the mechanism of non-homologous end joining (NHEJ), which is more prone to errors [1, 2]. The cellular choice of using HR or NHEJ is largely dependent on the phases of the cell cycle; NHEJ is present throughout the cell cycle, whereas HR predominates in the S and G2 phases, in order to ensure the high-fidelity preservation of genetic information [3]. If the repairing process does not occur correctly, the DNA injuries result in mutations and chromosomal aberrations which alter the cellular behavior and lead to cancer.

**Fig. 1:**
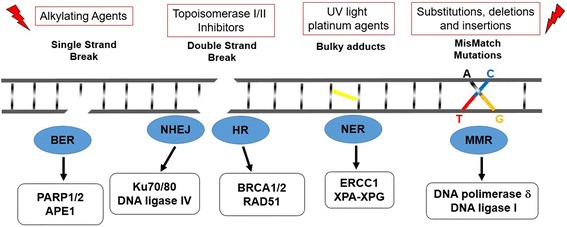
Diagram of targeted DDR pathways. In the *lower part* of the figure the DDR mechanisms and the related proteins involved are represented. In the *upper part* of the figure the targeting strategy for the corresponding defective DDR mechanisms are shown

Genes that encode for enzymatic or scaffolding proteins involved in the “core” DDR activities [BER, MMR, HR and NHEJ) are: XPA-XPG, RPA, ERCC1, DNA glycosylase, APE1, DNA polymerase β/δ/ε, XRCC1, DNA ligase 1/3, DNA ligase IV, Ku70/80, RAD50/MRE11/NBS1, BRCA1, BRCA2, and RAD51 (Fig. 1) [4–9].

Additionally, as a result of a computational analysis nearly 400 proteins have been identified in the regulation of the DDR processes [10–13], namely: the damage sensing kinases ATM/ATR, that activate a phosphorylation cascade signaling in response to the DSBs [14, 15]; DNA-PK, that cooperates with ATR and ATM to phosphorylate proteins involved in the DNA damage checkpoints and is required for NHEJ [16]; the kinases CHEK1 and CHEK2, that are responsible for slowing down the cell cycle progression to allow DNA repair [17]; and the nuclear phosphatase PTEN, that controls the transcription and the nuclear localization of the recombinase RAD51 [18–20]. Furthermore, ubiquitination, sumoylation, acetylation and methylation processes provide an additional layer of complexity targeting stability and efficiency of DDR proteins machinery [10, 12].

**Fig. 2:**
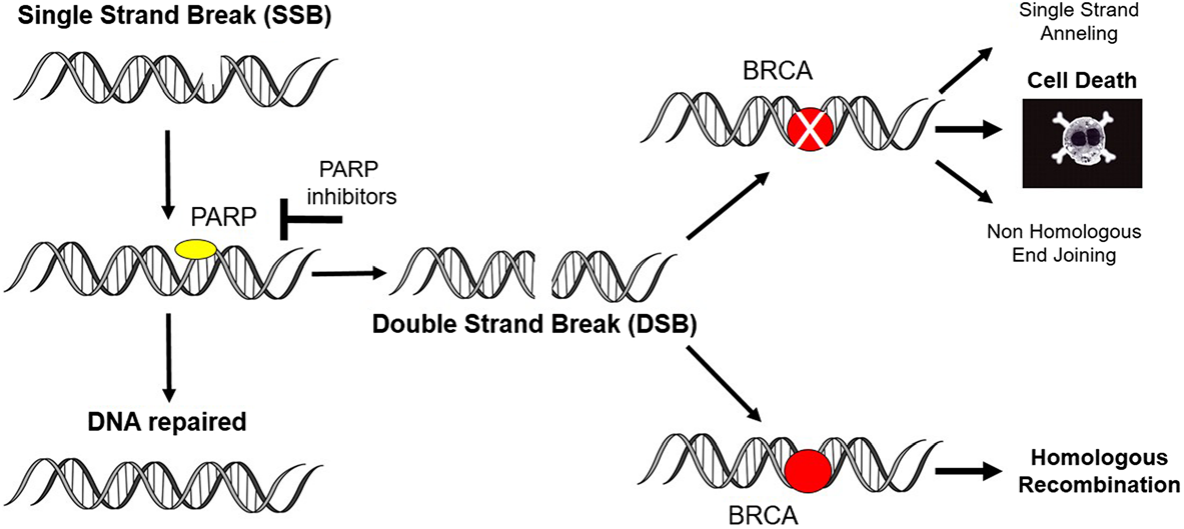
Synthetic Lethality of PARP-inhibitors in BRCA Tumors. Poly(ADP-ribose) polymerases (PARPs) repair DNA SSBs through the BER pathway. PARP inhibitors, such as olaparib, prevent repair of the SSBs, resulting in the generation of DNA DSBs. Cancer cells with a deficient homologous recombination (BRCA1/BRCA2 mutations) required for the repair of the DSBs do not compensate for the increased DNA damage caused by the inhibition of PARP enzymes and appear to be especially sensitive to treatment with these drugs

Since almost 56% of the identified 400 proteins are involved in multiple DDR pathways, a functional defect or loss of a single DDR protein may affect multiple DNA repair processes [11]. Defects in DDR seem to be positively selected in cancer cells to support the enhanced proliferation rate [21–23]. However, molecular alterations in the DNA repairing process make the cells more vulnerable to the pharmacological inhibition of the DNA repairing enzymes [24–30]. The concept of promoting the killing of cancer cells by simultaneously targeting cellular signals that cooperate and complement molecular defects to obtain cell death represents an anti-cancer strategy based on the concept of synthetic lethality (Fig. 2) [31–33].

Cancer cells defective in the DSBs repair molecules involved in DDR can be targeted specifically by blocking SSBs repair by inhibiting PARP enzymes [34–41].

## Main body

### PARP enzymes and defects in DNA damage response in cancer

The Poly ADP-ribose polymerase (PARP) family comprises 17 members including PARP1, PARP2, PARP3, tankyrases 1 and 2 (PARP5a and 5b), all of which have been identified on the basis of their homology in the catalytic domain [42–48]. The most studied protein of the PARP family is PARP1, a nuclear protein with enzymatic and scaffolding properties, that contains an amino-terminal DNA binding domain (DBD, a central auto-modification domain (AMD), crucial for protein-protein aggregation, and a carboxyl-terminal catalytic domain (CD). The activity of the PARP1 enzyme seems to be critical in the BER but also in the HR and NHEJ mechanisms [46, 49–51]. The PARP1 enzyme transfers the first ADP-ribose from nicotinamide adenine dinucleotide (NAD+) to the proteic residues (glutamate, aspartate and lysine) and generate an ADP-ribose unit chain (PAR), acting as a “writer” of a poly ADP-ribosylation [52, 53]. This process of “PARylation” occurs covalently on target proteins (transPARylation) and on the PARP enzyme itself (autoPARylation) producing a negative charge that alters the protein structure and function. The presence of PAR chains at the break-sites of DNA favors the non-covalent recruitment of DNA repair proteins like XRCC1, DNA ligase 3, DNA polymerase β, and the MRE11-Rad50-NBS1 (MRN) complex, for DNA resection and single strand formation, which in turn allow for RAD51 loading to initiate HR [43, 54–56].

PARP1 and PARP2 are the enzymes most extensively studied and are known to be stimulated by DNA damage, although PARP2 contributes to only 5–10% of the total PARP activity in response to DNA damage [57–60]. Tankyrases 1 and 2 are mostly involved in telomere and mitotic spindle-related functions, as well as in the regulation of Wnt signaling [61]. Overall, the PARP enzymes regulate DDR but also tumor growth and progression through transcriptional/epigenetic regulation and mRNA processing and stability [43, 62–68]. Thus, targeting PARP activity can cooperate and complement molecular defects of DDR that have been exploited therapeutically for different cancer treatments [69–72].

A wide variety of hereditary human cancers show germline alterations of genes involved in the DDR process. Germline mutations within BRCA1 carriers predispose to early onset of breast and ovarian cancer; mutations in BRCA2 genes lead to the late onset of different tumors (gastric, colon, pancreatic and melanoma) beyond breast, ovarian and prostate cancer; mutations in the ATM, NBS1, BLM, and WRN genes occur in lymphoma and leukemia; the RAD54 and CtIP genes are mutated in non-Hodgkin’s lymphoma and colon cancer; the MLH1 and MSH2 genes are mutated in hereditary non-polyposis colorectal cancer (HNPCC); the RAD51B gene is mutated in lymphoma and uterine leiomyoma and finally the RECQL4 gene is mutated in skin cancer and osteosarcoma [73–80]. Beside the DDR defects identified in hereditary human cancer, recent studies based on computational analysis have shown that almost 95% of DDR genes are mutated in coding regions at least in 15 different types of sporadic tumors [11], suggesting that mutations in DDR genes should be further investigated as possible driver mutations in cancer.

### The activity of PARP inhibitors: success and failure of PARPi in Clinical Trials

The use of PARP enzyme inhibitors in cancer cells which are defective for BRCA1 and BRCA2, two proteins that localize the RAD51 recombinase to the sites of damaged DNA and promote HR repair, represent the best and most successful synthetic lethal approach in cancer therapy.

The PARP inhibitors bind the catalytic domain of the PARP protein, mostly as antagonists of the PARP cofactor β-NAD+. Because of the binding of the inhibitors, the PARP enzymes could be inhibited in the catalytic activity, with the final result of converting SSBs into DSBs and determining cell death in DSB repair deficient cells [81–88].

There are a total of nine drugs related to PARP targeting in the pipeline of drug development, some of them having a selective activity on PARP1 and PARP2, others affecting both PARP1 and PARP2 [89].

AG-014699 Rucaparib (Clovis/Pfizer), used for intravenous and oral administration, is the first inhibitor that has been introduced in clinical trials in association with chemotherapy and is now in Phase III for maintenance treatment after chemotherapy [90–92]. AZD2281 Olaparib (kuDOS/LynparzaTM, AstraZeneca), used for oral administration, is the first inhibitor identified as single antitumoral agent in cancer associated with the BRCA1 or BRCA2 mutations [93, 94]. Notably, in December 2014 Olaparib was approved by the US Food and Drug Administration (FDA) for its use in pretreated ovarian cancers with defective BRCA genes and by the European Medicines Agency (EMEA) for maintenance therapy in BRCA-mutated/platinum sensitive ovarian cancer patients. ABT-888 Veliparib [Abbvie], used for oral administration, is now in Phase III evaluation in combined therapy in advanced or recurrent solid tumors, also because of its property to reach the Central Nervous System (CNS) [95, 96]. BSI-201 Iniparib (BiPar/Sanofi), used for intravenous administration, was the first inhibitor that has entered Phase III studies and, besides the discrepancies between the phase II and III studies, has shown antitumor activity mostly in combined therapy with gemcitabine and carboplatinum [97–99]. MK-4827 Niraparib (Merck/Tesaro), used for oral administration, is a potent inhibitor of PARP1 and PARP2 and is currently being tested in Phase III clinical trials as maintenance therapy in ovarian cancer and as a treatment for breast cancer [100–102]. BMN-673 (BioMarin), used for oral administration, is more potent than Olaparib and is used in combined therapy in Phase III evaluation [103, 104]. Finally, a few drugs developed more recently like CEP-9722/8983 (Cephalon) and E7016 or E7449 (Eisai/MGI Pharma) are used for oral administration and are now being tested in Phase I toxicity studies in combined therapy [88, 105, 106]

By limiting the DDR competence, PARP inhibitors complement the mechanisms of action of chemotherapy and radiotherapy. Thus, as chemosensitizer, PARPi, has entered clinical assessment in combination with temozolomide [90, 107], DNA crosslinkers (e.g. cisplatin) [108], or cleave the sugar-phosphate backbone (e.g. bleomycin) [109]. Thus, several clinical trials with different PARP inhibitors have been conducted and are still ongoing to test the efficacy of PARPi as a single agent or in combination with radiotherapy and/or chemotherapy [https://clinicaltrials.gov/]. Clinical trials that are in progress to test PARPi efficacy have been recently summarized by Lord and Ashworth [110].

In 2005 a Phase I clinical trial assessing Olaparib as a single agent showed an objective response rate (ORR) of 47% and a disease control rate (DCR) of 63% in patients with BRCA mutations [94]. Next, Phase II studies confirmed the efficacy of Olaparib as a single agent in breast and ovarian cancer patients with BRCA mutations [111]. Significantly, the best response to Olaparib has been reported in ovarian cancer patients with BRCA mutations who have demonstrated a sensitivity to platinum treatment; patients who had a platinum-sensitive disease were Olaparib responsive with a rate of 69%, whereas those who were platinum-refractory had a response rate of 23% [111]. Thereafter, clinical trials were designed to define both the utility of Olaparib as a single agent in chemotherapy-refractory tumors, and as maintenance therapy in platinum-sensitive diseases. Carriers of BRCA mutations, responsive to previous platinum therapy, have shown better outcomes in PFS (8.4 months vs 4.8 months) with the Olaparib maintenance therapy [112–114].

Currently, Phase III clinical trials are in progress aimed at assessing the use of several PARP inhibitors (e.g. Olaparib, Niraparib, Recuparib, Veliparib, and BMN-673) in the maintenance therapy of platinum treated ovarian and breast cancer, as single agents in therapeutic settings of adjuvant and standard-of-care chemotherapy for advanced diseases. Additionally, Phase III clinical trials are ongoing to assess the efficacy of PARP inhibitors in combined treatments, in therapeutic settings of neoadjuvant strategy and in advanced diseases [https://clinicaltrials.gov/].

The positive results obtained in breast and ovarian and prostate cancer patients with BRCA mutations [111–115] have encouraged the use of PARP inhibitors in tumors with BRCA-like features (*BRCAness phenotype*). Thus, the efficacy of PARPi has been evaluated in Triple Negative Breast Tumors and High-Grade Serous Ovarian Cancer (TNBC/HGS-OVCa) that exhibited a 60-gene signature in common with BRCA mutated tumors [110, 116–121]. Moreover, clinical correlation suggested that the BRCA status conferred sensitivity to platinum chemotherapy and this can be used as a marker for HR defects to predict PARP-inhibitors sensitivity [122].

### Predicting PARPi sensitivity beyond BRCA1/2 mutations and “BRCAness”

The identification of biomarkers that can predict the PARP inhibitor sensitivity of cancer cells is urgently required. Germline BRCA mutations are recognised as the molecular targets for PARPi sensitivity in breast, ovarian and prostate cancer. However, it is noteworthy that PARPi efficacy has also been reported in non-BRCA related tumors [123]. Thus, PARP inhibitors may have a utility beyond the relatively small proportion (5–10%) of cancer patients carrying BRCA mutations [102, 124].

Tumors displaying a DNA repair dysfunction, regardless of the leading genetic lesion, might exhibit a BRCA-like behavior, according to the concept of “BRCAness” [110, 116], and might therefore benefit from PARPi treatment [122, 125–128]. However, the predictive value of a “BRCAness” signature, besides BRCA1 and BRCA2 mutations, still requires prospective large-scale clinical validation before entering conventional clinical practice. Genomic alterations, gene mutations or functional loss of proteins involved in DDR mechanisms, such as ATM, ATR, CHEK1, CHEK2, DSS1, RAD51, MRE11A/NBS1, Fanconi anemia complementation group (FANC family of genes), EMSY, PALB2, XRCC2, XRCC3, or PTEN, could represent predictive markers in cancer patients to tailor a personalized treatment with PARP inhibitors [129–141].

Nevertheless, additional molecules appear to be able to influence the sensitivity to PARPi, namely the gene fusions TMPRSS2:ERG, detected in more than 50% of prostate cancers, and the EWSR1:FLI1 translocation, detected in Ewing’s sarcoma in more than 90% of patients [142, 143].

To date, a few additional proteins have been proposed as novel predictive biomarkers of PARP inhibitor sensitivity like cyclin dependent kinase CDK12, the excision repair cross-complementation group 1 ERCC1, and the proapoptotic protein CCDC6.

CDK12 attenuation in the high-grade serous ovarian cancer model [HGS-OVCa] is sufficient to confer a sensitivity to PARP1/2 inhibition [144, 145].

ERCC1 low expression in the NSCLC model is sufficient to determine a synergistic effect with PARP inhibition [146, 147].

CCDC6 loss or low expression in different cancer models impairs RAD51 foci formation, limits γH2AX foci formation by modulating the activity of the histone phosphatase PP4C and sensitizes the cancer cells to PARPi treatment [148–151].

Moreover, kinases, such as CDK5, MAPK12, PLK3, PNKP, STK22c, STK36, and deubiquitinases, such as USP1 and USP11, can produce synthetic lethal effects with PARPi in genetic perturbation screens performed following different approaches [152–157].

In Table 1 we report the altered genes which have been described so far to confer PARPi sensitivity in different in vitro and in vivo cellular models.

**Table 1:** Genetic alterations that predict PARPi sensitivity

Altered gene	PARP inhibitors	in vitro/in vivo systems	Study
BRCA2	NU1025 and AG14361	Chinese hamster cell V-C8 and V-C8 + B2 (BRCA2 defective and BRCA2 complemented) Human breast cancer cells MCF-7 or MDA-MB-231 (BRCA2 siRNA)	Bryant H. et al., Nature 2005. .
	KU0058684 and KU0058948	Mouse ESC (lacking BRCA2 wt) HeLa (RNAi)	Farmer H. et al, Nature 2005 McCabe N. et al., Cancer Res. 2006
	Olaparib (AZD2281)	Ovarian cancer patients ClinicalTrial **NCT00753545** Prostate cancer patients ClinicalTrial, **NCT01682772**	Ledermann J. et al., N Engl J Med. 2012. Mateo J. et al., N. Engl. J. Med. 2015.
ATM	KU0058684 and KU0058948 Olaparib (AZD2281)	HeLa (RNAi) Prostate cancer patients ClinicalTrial, **NCT01682772**	McCabe N. et al., Cancer Res. 2006. Mateo J. et al., N. Engl. J. Med. 2015.
ATR	KU0058684 and KU0058948	HeLa (RNAi)	McCabe N. et al., Cancer Res. 2006
FANC A/F	KU0058684 and KU0058948 Olaparib (AZD2281)	Mouse fibroblast (FANC KO) Prostate cancer patients ClinicalTrial, **NCT01682772**	McCabe N. et al., Cancer Res. 2006 Mateo J. et al., N. Engl. J. Med. 2015.
CHK2	KU0058684 and KU0058948 Olaparib (AZD2281)	HeLa (RNAi) Prostate cancer patients ClinicalTrial, **NCT01682772**	McCabe N. et al., Cancer Res. 2006 Mateo J. et al., N. Engl. J. Med. 2015.).
BRCA1	KU0058684 and KU0058948 Olaparib (AZD2281)	Mouse ESC (lacking BRCA2 wt) HeLa (RNAi) Ovarian cancer patients ClinicalTrial **NCT00753545** Prostate cancer patients ClinicalTrial, **NCT01682772**	Farmer H. et al, Nature. 2005. McCabe N. et al., Cancer Res. 2006. Ledermann J. et al., N Engl J Med. 2012. Mateo J. et al., N. Engl. J. Med. 2015.
PALB2	Olaparib (AZD2281) BMN 673	Human fibroblast EUFA1341 (express PALB2 mutant) Wilms tumor KT-10 cells (express truncated PALB2)	Buisson R. etal., Nat Struct Mol Biol. 2010. Smith MA. et al., Pediatr Blood Cancer. 2015.
RAD51B/C	KU0058684 and KU0058948 Olaparib (AZD2281)	HeLa (RNAi) Prostate cancer patients ClinicalTrial, **NCT01682772**	McCabe N. et al., Cancer Res. 2006. Mateo J. et al., N. Engl. J. Med. 2015.
RAD54	KU0058684 and KU0058948	Mouse ESC (lacking Rad54 wt)	McCabe N. et al., Cancer Res. 2006.
ERCC1	olaparib (AZD-2281), niraparib (MK-4827), BMN 673 olaparib (AZD-2281), veliparib (ABT-888)	Non Small Cell Lung Cancer A549 (ERCC1 deficient clones) Non Small Cell Lung Cancer HCC827, PC9 (ERCC1 low expression)	Postel-Vinay S. et al., Oncogene 2013. Cheng H. et al., Carcinogenesis 2013.
*CtIP*	BMN 673 KU0058948	Myeloid leukemia cell K562 (expressing *CtIP-T)*	Gaymes TJ. et al., Haematologica. 2013.
MRE11	BMN 673 KU0058948	Hystiocitic Linphoma U937 cell (expressing MRE11-Δ57)	Gaymes TJ. et al., Haematologica. 2013.
NBS1	KU0058684 and KU0058948	Human immortalized fibroblast	McCabe N. et al., Cancer Res. 2006.
DSS1	KU0058684 and KU0058948	HeLa (RNAi)	McCabe N. et al., Cancer Res. 2006.
RPA1	KU0058684 and KU0058948	HeLa (RNAi)	McCabe N. et al., Cancer Res. 2006.
PTEN	(KU0059436) Olaparib	Colorectal tumour cell HCT116 (truncated PTEN) endometroid adenocarcinoma cells HEC1A (truncated PTEN) Breast, prostate, melanoma, glioma and bladder cells HCC70, MDA-MB-468, PC3, RPMI-7951, A172, UM-UC3 and (PTEN deficient expression)	Mendes-Pereira, A. et al., EMBO Mol. Med. 2009.
	(KU0059436) Olaparib	Prostate cancer patients ClinicalTrial, **NCT01682772**	Mateo J. et al., N. Engl. J. Med. 2015.
*ETS/ERG*	(KU0059436) Olaparib MK-4827	Solid tumors ClinicalTrial, **NCT00777582** Solid tumors ClinicalTrial, **NCT00749502**	Brenner JC. et al, Cancer Res. 2011.
XRCC2/XRCC3	(3-AB) (ISQ) (NU1025) (AG14361)	Chinese hamster ovary cell irs1/irs1SF	Bryant H. et al., Nature 2005.
CDK1	AG14361 and AG014699	Non Small Cell Lung Cancer NCI-H1299/A549 CDK1 (RNAi)	Johnson N. et al., Nat Med. 2011.
CDK12	veliparib (ABT-888)	Ovarian cancer OVCAR-3, OVCAR-5, OVCAR-8 CDK12 (RNAi)	Joshi, P.M. et al., J. Biol. Chem. 2014. Bajrami,I. . et al., Cancer Res. 2014.
	olaparib (AZD-2281)	Ovarian cancer PEO1, OV56, COV504; OV90 cells CDK12-(RNAi)	
	(KU0059436) Olaparib	Prostate cancer patients ClinicalTrial, **NCT01682772**	Mateo J. et al., N. Engl. J. Med. 2015.
CDK5	KU0058948	Breast cancer CAL51 cells CDK5 (RNAi)	Turner, N.C. et al., EMBO J. 2008.
PLK3	KU0058948	Breast cancer CAL51 cells PLK3 (RNAi)	Turner, N.C. et al., EMBO J. 2008.
PNKP	KU0058948	Breast cancer CAL51 cells PNKP (RNAi)	Turner, N.C. et al., EMBO J. 2008.
STK22C	KU0058948	Breast cancer CAL51 cells STK22C (RNAi)	Turner, N.C. et al., EMBO J. 2008.
STK36	KU0058948	Breast cancer CAL51 cells STK36 (RNAi)	Turner, N.C. et al., EMBO J. 2008.
USP1	KU0058948	Breast cancer CAL51 cells USP1 (RNAi)	Turner, N.C. et al., EMBO J. 2008.
USP11	olaparib (AZD-2281)	Bone osteosarcoma U2OS cell USP11 (RNAi)	Wiltshire TD. et al., J Biol Chem. 2010.
CCDC6	olaparib (AZD-2281),	Non Small Cell Lung Cancer H1975 cells CCDC6 (RNAi)	Morra F. et al., Int J Cancer 2015.

The complexity of the existing crosstalk between DNA repair pathways suggests that a single biomarker may not be sufficient to predict the benefit of PARP inhibitor therapies. Therefore, DNA microarrays, real-time quantitative reverse transcriptase [qRT]-PCR, protein microarrays, mass spectrometry, immunohistochemistry and immunofluorescence assays represent powerful tools in order to identify predictive biomarkers measured at baseline or in progress of therapy in cancer patients enrolled on PARPi clinical studies.

### Assays that can measure HR proficiency and PARP activity in vivo

The development of novel assays that are able to predict the HR proficiency in cancer cells represents an important challenge in the design of a personalized therapy. Several assays that can evaluate the HR proficiency in cancer cells need to be validated in prospective clinical trials for their value to predict the response to targeted therapies, Since genomic instability may represent a lifetime record of DNA repair deficiency, the genomic structural rearrangement signatures, identified in functional BRCAness, should be examined in tumor types other than ovarian and triple-negative breast cancers. [158–164].

It has been reported that the measure of telomeric allelic imbalances (NtAl), that counts the number of subtelomeric regions with allelic imbalance,in combination with the measure of loss of heterozygosity (ARD-LOH), that measures the number of regions with LOH which are larger than 15 Mband with the measure of large scale transition (LST), that counts the number of chromosomal breaks between adjacent regions of at least 10 Mb, can identify in 15 different tumor types a genomic scar signature in SNP array data and allow the selection of DNA repair-deficient cancers, to candidate cancer patients to platinum chemotherapy and PARPi [158]. On this basis Myriad Genetics used SNP profiling to develop a HR deficiency (HRD) assay which combines the mentioned three different DNA-based metrics of genomic instability [165]. The HDR assay can be performed using DNA extracted from FFPE tumor tissues and thus has been translated into clinic for perspective studies (ARIEL2 NCT#01891344)

Furthermore, a biological rationale driven (mutations in BRCA1/2) genomic instability score has been developed by integrating somatic mutations and copy number changes reported in the TCGA of 325 ovarian cancers. The identified score has been demonstrated to correlate with homozygous deletion of core HR genes in 67 HR deficient non BRCA samples compared to 152 control samples. The identified score has been also correlated with the outcome of response to treatment of platinum in ovarian cancer patients [166].

Several studies support the use of panel testing for a comprehensive analysis of mutations, expression changes of multiple genes in prospectively designed trials for the selection of patients likely to respond to platinum and PARPi. However, there is currently no gold standard method of testing for DDR cancer cell proficiency and cancer sensitivity to platinum or PARPi.

Assessing the number of nuclear foci of RAD51, FANCD2 and γH2AX may help to establish DDR cellular competence, before, during and after treatment with IR and PARP inhibitors, to predict tumor sensitivity or acquired resistance to treatments.

Therefore, the detection of RAD51 foci by IHC and IF, in ex vivo samples or in tumor biopsies during neoadjuvant therapy, may be predictive of HR defects and sensitivity to PARPi, given that PARP inhibition or loss results in an increasing RAD51 foci formation in HR intact cells [167]. This approach of RAD51 foci formation has been applied in ex vivo samples in primary ovarian cancer cultures to predict sensitivity toRucaparib, showing a negative predictive value of 100% and a positive predictive value of 93% [168]. The analysis of RAD51 foci formation in FFPE samples of breast cancer biopsied after neoadjuvant therapy also has appeared to be predictive of a response to chemotherapy [169].

The FANCD2 foci formation may be assayed to predict the sensitivity of cancer cells to cisplatin and PARPi, given that PARP inhibitors, chemotherapy and radiotherapy induce FANCD2 nuclear foci formation [170–172].

Additionally, a high-throughput screening system based on the IF of γH2AX (Rapid Automated Biodosimetry Tool) may help to screen patients sensitive to PARPi treatment, given that PARP inhibitors increase γH2AX foci in Circulating Tumor Cells [173–175]. Recently, the evaluation of RAD51 and γH2AX nuclear foci in ex vivo samples, as well as of the PARP activity, has been applied in some clinical studies [168, 176, 177].

Ongoing clinical trials with PARPi [ABT-888], either as a single agent or in combination therapy, aim to identify suitable patients for PARPi sensitivity, beside BRCA mutations, that show HR or MMR deficiency (NCT01237067, NCT02660034, NCT02576444, NCT02286687, NCT01891344, NCT02354131) by measuring γH2AX and FANCD2 foci formation in FFPE tumor samples [167, 172, 177] (NCT01017640; NCT01251874).

Tumor cells defective in the HR process might also show a compensatory induction of PARP expression. However, PARP1 protein levels do not differ between isogenic pairs of HR deficient and proficient cancer cells [178]. An enhanced PARP1 expression does not mean an enhanced PARP activity [179–182]. Since the PARP activity is associated with the levels of PAR, the detection of low levels of PAR may indicate an intrinsic low activity of PARP with a limited potential of PARP inhibitor efficacy. Therefore, the prediction of PARPi efficacy could possibly be evaluated by assays that quantify the intrinsic PARP activity in cancer cells. An Enzyme Linked Immunosorbent Assay (ELISA) may be applied for a quantitative and sensitive assessment of PAR (poly[ADP]ribose) polymer levels in tumor specimens and in peripheral blood to assess the enzyme proficiency and to predict PARPi sensitivity [176].

## Conclusions

Emerging data suggest that PARP inhibition is a potentially important strategy for managing a significant subset of tumors. The discovery of both germline and somatic DNA repair deficiencies in different cancer patients, together with the development of PARP inhibitors that can kill cancer cells with these defects, is a potent example of targeting therapy to molecularly defined tumor subtypes. The assessment of genomic instability assays and of nuclear foci status, together with the levels of DNA repairing proteins may predict the outcomes of PARPi treatment in different cancer types [183–187]. In the near future, the systematic evaluation of PAR levels, γH2AX, FANC and RAD51 foci with genomic instability features in tumor biopsies before, during and after treatment might help to identify patient populations who can be classified as responders or non-responders to PARP inhibitors. However, the heterogeneity of the tumor could always limit the evaluation of specific biomarkers, especially when the analysis is performed on a small amount of tissue (tissue microarrays, TMA) by IHC or FFPE.
